# Associations of maternal pre-pregnancy body mass index and gestational weight gain with risk of offspring neurodevelopment at 2 years: A Chinese birth cohort study

**DOI:** 10.3389/fped.2023.1165743

**Published:** 2023-04-18

**Authors:** Xiaohan Dong, Aifen Zhou

**Affiliations:** ^1^ Department of Obstetrics and Gynecology, Wuhan Children’s Hospital (Wuhan Maternal and Child Healthcare Hospital), Tongji Medical College, Huazhong University of Science & Technology, Wuhan, China; ^2^ Institute of Maternal and Child Health, Wuhan Children’s Hospital (Wuhan Maternal and Child Healthcare Hospital), Tongji Medical College, Huazhong University of Science & Technology, Wuhan, China

**Keywords:** overweight, pre-pregnancy, gestational weight gain, obese, neurodevelopment, body mass index

## Abstract

**Introduction:**

In recent decades, there has been a surge in both obesity and developmental impairments. Only a few research have looked at the relationship between gestational weight growth and pre-pregnancy BMI in mothers and the neurobehavioral development of their infants. The current research investigates the associations among maternal pre-pregnancy BMI, GWG, and the risk of child neural development at 2 years of age depending on a Chinese birth prospective study.

**Methods:**

The study population was 3,115 mother-infant pairs were registered in the Wuhan Health Baby cohort between September 2013 and October 2018, and data from this cohort was used in this investigation. The Chinese classification was used to group maternal BMI before conception. Based on the 2019 Life Cycle Project-Maternal Obesity and Childhood Outcomes Study Group, categories for GWG were created. The outcome was an assessment of child neural development at age 2 which was measured by employing a Chinese translation of the Bayley scales (BSID-CR). The multivariate regression models were used to calculate the beta (*β*) coefficients and 95% confidence intervals (CIs) for estimating the associations between continuous Bayley scores and maternal pre-pregnancy BMI categories, as same as in GWG categories.

**Results:**

Infants of overweight and obese moms exhibited lower MDI scores than those of mothers with normal pre-pregnancy BMI (*β *= −2.510, 95%CI*** ***= −4.821 to −0.200) in the entire sample. Meanwhile, we find among the normal pre-pregnancy BMI mothers, infants of inadequate GWG mothers had lower MDI scores (*β *= −3.952, 95%CI*** ***= −7.809 to −0.094) compared with the referenced adequate GWG mothers, as well as the infants of excessive GWG mothers among the underweight pre-pregnancy BMI mothers (*β *= −5.173, 95%CI*** ***= −9.803 to −0.543). The PDI scores of the infants were not affected by the maternal pre-pregnancy BMI or GWG.

**Conclusion:**

For Chinese babies aged 2 in this nationally representative sample, aberrant pre-pregnancy BMI and GWG can impair infants’ mental development, but not psychomotor development. Such results are significant given the incidence of overweight and obesity as well as the long-term effects of early brain development. In this study we found optimal GWG recommendations proposed by 2019 Life Cycle Project-Maternal Obesity and Childhood Outcomes Study Group were more suitable for Chinese women than 2009 Institute of Medicine(IOM) guidelines. Additionally, women should be given general advice on how to achieve their ideal pre-pregnancy BMI and GWG.

## Introduction

Negative health consequences for mothers and babies are strongly correlated with the incidence of overweight and obesity among women of reproductive age (1–3). Recent epidemiologic studies have shown that mother pre-pregnancy BMI and gestational weight gain (GWG) are risk factors for impaired infant neurodevelopment that may be modifiable (4–9). Potential causes of aberrant foetal brain development and deficiencies in cognitive, linguistic, and motor abilities include hormonal and inflammatory-induced alterations (10–12), as well as excessive or inadequate nutritional consumption during pregnancy (13, 14). According to animal research, significant amounts of inflammatory cytokines (15, 16) that influence brain development and later neurodevelopmental functioning can result from an overweight condition with increased GWG. Contrarily, some research indicates that adequate GWG may be able to mitigate the negative effects of greater BMI numbers on neonate/child neurodevelopment (7, 17–20). The effect for infants at 2 years of age in the Chinese population was rarely appreciated, despite the causal association between pre-pregnancy BMI, GWG, and worse neurobehavioral development in newborns being shown. Clear ethnic disparities in BMI and GWG have been seen in people with various genetic backgrounds, living situations, and lifestyles, particularly in some US population-based cohort studies (21, 22). As GWG levels differ with ethnicity, different BMI classes may be also connected with them (23). The relationships between the risk of child neurodevelopment and BMI and GWG in various ethnic communities must therefore be reproduced and verified. This study looked into any connections between maternal pre-pregnancy BMI, GWG, and the likelihood of adverse infant neural advancement in the Chinese.

## Materials and methods

### Participants of study

The Wuhan Health Baby cohort, which was carried out in Wuhan, China, and was intended to ascertain the effect of maternal exposures on child health, was the source of the individuals. This research included pregnant women who underwent their initial antenatal checkup at Wuhan Maternal and Child Healthcare Hospital between September 2013 and October 2018. The pregnant person must meet the requirements at enrollment in order to be eligible: Residents of Wuhan City, Singleton Gestation, Under Gestational 16 Weeks, Prepared to Fill Out Questionnaires, Preparation for Prenatal Checkups, and Preparation for Delivery at Study Healthcare. 3,598 mother-child couples were included. Participants with incomplete pregnancy and delivery data (*n* = 17) and people who had unqualified neurodevelopmental assessment results at age 2 (*n* = 392) were eliminated from the group. The final research group included 3115 mother-child couples after removing the children who experienced asphyxia (*n* = 6), preterm labour (*n* = 53), and foetal growth retardation (*n* = 15).

### Ethics statement

Proof of permission is available upon request. The medical ethics committee at the Wuhan Maternal and Child Healthcare Hospital (Wuhan Medical & Healthcare Center for Women and Children), Tongji Medical College, Huazhong University of Science and Technology has approved this work for research ethics. When participants visited the hospital for the first time for a prenatal examination, they were all asked to sign written informed consent forms.

### BMI and GWG

Maternal pre-pregnancy BMI and GWG were exposed. Maternal self-reports of pre-pregnancy weight and height were collected at the start of the study. Weight (kg)/height (m)2 was used to compute pre-pregnancy BMI, which was then classified using the Chinese BMI classification standard (24), which is more appropriate for the Chinese population and several other Asian Pacific populations (25) than the World Health Organization (WHO) standard. Women were thus split into four groups based on the categories of the Working Group on Obesity in China: underweight (BMI < 18.5 kg/m^2^), normal weight (18.5 kg/m^2 ^≤ BMI < 24.0 kg/m^2^), overweight (24.0 kg/m^2 ^≤ BMI < 28.0 kg/m^2^), and obese (BMI ≥ 28.0 kg/m^2^) (24). Because there were only 66 people in the “obesity” group, the category was amalgamated with the “overweight” category and is now referred to as the “overweight/obese” classification. The three BMI categories of “underweight,” “normal weight,” and “overweight/obese” were then statistically compared, using women with normal BMIs (“normal weight”; 18.5–24.0 kg/m^2^) as the reference group.

Maternal GWG was estimated as the discrepancy between the most recent menstrual cycle’s self-reported weight and the last weight that was clinically measured and recorded prior to or at delivery (26). GWG categories of inadequate, sufficient, and excess were developed based on the 2019 Life Cycle Project-Maternal Obesity and Childhood Outcomes Study Group (1). For women classified as underweight, adequate weight increase ranged from 14.0 kg to less than 16.0 kg (BMI < 18.5 kg/m^2^); 10.0 kg to less than 18.0 kg for normal weight (BMI, 18.5–24.9 kg/m^2^); 2.0 kg to less than 16.0 kg for overweight (BMI, 25.0–29.9 kg/m^2^); 2.0 kg to less than 6.0 kg for obesity grade 1 (BMI,30.0–34.9 kg/m^2^); For obesity grade 2 (BMI, 35.0–39.9 kg/m^2^), weight reduction or growth of 0 kg to less than 4.0 kg is required, and for obesity grade 3 (BMI, 40.0 kg/m^2^), weight gain of 0 kg to less than 6.0 kg is required. During the analysis, women with adequate GWG served as the reference group.

### Children neurodevelopment

To evaluate the neurodevelopment of babies at a mean age of 2 years (27), we utilised the Chinese revision of the Bayley Scale of Infant Development (BSID-CR). The Bayley Scales of Infant Development (BSID) was converted into the BSID-CR after some additional localization revisions (27). The BSID-CR performed satisfactorily and was implemented extensively in China (27). Highly trained psychiatrists who were unaware of the infants’ exposure data conducted the examinations. The entire testing procedure was carried out in accordance with hospital protocol and was captured on film. The accompanying main indicators were primarily included in the BSID-CR measurement results: (1) The Mental Development Index (MDI), which measured the cognitive growth of infants and included measures of language, generalisation, classification, memory, and social skills; (2) The Psychomotor Development Index (PDI), which measured the psychomotor growth of infants and included measures of muscle coordination and gross and fine manipulation skills. With a mean of 100 and a standard deviation of 15, all raw scores were standardised to get normative scores (27).

### Covariates

After delivery, skilled nurses conducted face-to-face interviews with the participants to gather socioeconomic and demographic data, such as the mother’s age, education level, family’s yearly income, and lifestyle factors (e.g., passive smoking during pregnancy). Medical records were used to gather information on other aspects of the mother’s pregnancy, including parity, delivery method, date of last menstrual period (LMP), gestational diabetes mellitus (GDM), hypertensive disorders of pregnancy (HDP), model of delivery, birth date, and the characteristics of the infant (such as gender, gestational age at birth, and birth weight). The birth date less the LMP date was used to calculate the gestational age (weeks).

### Statistical analysis

The relationship between the mother’s pre-pregnancy BMI, GWG, and continuous Bayley ratings is assessed using multivariable linear regression using beta (*β*) coefficients and 95% confidence intervals (CIs). Potential confounding factors included demographic factors (maternal age, gender, and education), lifestyle factors (passive smoking during pregnancy, family yearly income), obstetric factors (parity, delivery mode, gestational age, and birth weight), and medical conditions (gestational diabetes mellitus (GDM), hypertensive disorders of pregnancy (HDP). In the current investigation, two models were examined after potential confounders were taken into account. Model 1 was modified to account for maternal characteristics such as maternal age, parity, education, GDM, HDP, annual family income, and passive smoking while pregnant. Model 2 underwent further adjustments for neonatal variables such as delivery method, gender, gestational age, and birth weight. For this model, we looked at the data to see if there was any evidence of a multiplicative statistical interaction among pre-pregnancy BMI status and adequate gestational weight growth. The regression models automatically removed cases with missing confounder data. The pre-pregnancy BMI or appropriate GWG were employed as the reference in all regression model results. Further to investigate the association between GWG and neurodevelopment in various pre-pregnancy BMI categories, we performed a stratification analysis and separated the samples into subgroups based on their prenatal BMI classes.

We did a sensitivity analysis because the data were primarily dependent on the parents’ self-reports in order to address the probable misclassification of GWG and BMI. We conducted the stratified analysis after adopting the World Health Organization (WHO) BMI classifications (28) and IOM GWG recommendation (29) as alternative criteria in the sensitivity analysis. The WHO BMI range for being underweight was the same as the Chinese range, but it was wider for the other classes. The 2019 Life Cycle Project-Maternal Obesity and Childhood Outcomes Study Group suggestion for excessive weight gain was narrower than the range of the IOM recommendations for adequate weight gain, which were wider in the underweight and obese pre-pregnancy BMI categories and narrower in the normal weight and overweight pre-pregnancy BMI categories.

The SPSS programme (version 26.0, IBM SPSS Inc., Chicago, IL) was used for all statistical analyses. Based on two-tailed tests, *P* < 0.05 indicated significant differences for key variables, and *P* < 0.1 indicated substantial associations. Counts and percentage are used to express categorical variables. If continuous variables are regularly distributed, they are expressed as means (SDs), otherwise as medians (interquartile ranges) if the dispersion is skewed.

## Results

In Table 1, it is evident that maternal pre-pregnancy BMI was related to maternal age, parity, GDM, HDP, gestational age, birth weight, and MDI scores, whereas GWG was related to maternal age, mother education, family yearly income, parity, GDM, HDP, gestational age, and birth weight. Before becoming pregnant, the majority of mothers (66.5%) had BMIs that were within the normal range, and only a tiny percentage (19.7%) or 13.8% were underweight or overweight. 9.9% of women had inadequate GWG, 51.0% had appropriate GWG, and 39.1% had excessive GWG, based on the 2019 Life Cycle Project-Maternal Obesity and Childhood Effects Study Group recommendations. The median (25th–75th) maternal age was 28 (27–31) years, with a range of 19 to 43 years. Mothers were primiparous in the great majority (81.6%) and had college experience (80.4%). Only a small percentage of mothers (5.7%) either smoked themselves or did so while they were pregnant. In addition, the ratio of male-to-female newborns was almost 1:1 (52%–48%). 50.3% of the babies were delivered *via* caesarean section. 3357.5(390.6) g, 107.97(22.22), and 107.83(18.34), respectively, were the mean (SD) birth weights, MDI scores, and PDI scores. The median gestational age (25th–75th percentile) was 39.4(38.9–40.1)weeks.

**Table 1:** Features of mothers and babies based on pre-pregnancy body mass index (BMI) and gestational weight gain (GWG) (*n**** ***= 3,115).

	Total sample (*n**** ***= 3,115),%	Pre-pregnancy BMI category			Gestational weight gain category		
		Underweight (*n**** ***= 614),%	Normal weight (*n**** ***= 2,071),%	Overweight/obese (*n**** ***= 430),%	Inadequate (*n**** ***= 309),%	Adequate (*n**** ***= 1,589),%	Excessive (*n**** ***= 1,217),%
Maternal age (years)^a,b,d^	28 (27–31)	27 (26–39)	28 (27–31)	30 (27–33)	28 (26–31)	29 (27–32)	28 (26–30)
Maternal education^b^							
≤High school	611 (19.6)	152 (4.9)	373 (12.0)	86 (2.8)	63 (2.0)	281 (9.0)	267 (8.6)
≥college	2,504 (80.4)	462 (14.8)	1,698 (54.5)	344 (11.0)	246 (7.9)	1,308 (42.0)	950 (30.5)
Family yearly income (¥)^b^							
≤50,000	410 (13.2)	99 (3.2)	261 (8.4)	50 (1.6)	44 (1.4)	180 (5.8)	44 (1.4)
50,000–100,000	1,164 (37.4)	218 (7.0)	772 (24.8)	174 (5.6)	116 (3.7)	590 (18.9)	116 (3.7)
≥100,000	1,541 (49.5)	297 (9.5)	1,038 (33.3)	206 (6.6)	149 (4.8)	819 (26.3)	149 (4.8)
Passive smoking during pregnancy							
Yes	179 (5.7)	34 (1.1)	120 (3.9)	25 (0.8)	23 (0.7)	94 (3.0)	62 (2.0)
No	2,936 (94.3)	580 (18.6)	1,951 (62.6)	405 (13.0)	286 (9.2)	1,495 (48.0)	1,155 (37.1)
Parity^b^							
Primiparous(0)	2,542 (81.6)	542 (17.4)	1,688 (54.2)	312 (10.0)	244 (7.8)	1,231 (39.5)	1,067 (34.3)
Multiparous (≥1)	573 (18.4)	72 (2.3)	383 (12.3)	118 (3.8)	65 (2.1)	358 (11.5)	150 (4.8)
GDM^a,b^							
Yes	250 (8.0)	18 (0.6)	168 (5.4)	64 (2.1)	33 (1.1)	157 (5.0)	60 (1.9)
No	2,865 (92.0)	596 (19.1)	1,903 (61.1)	366 (11.7)	276 (8.9)	1,432 (46.0)	1,157 (37.1)
HDP^a,b^							
Yes	61 (2.0)	4 (0.1)	30 (1.0)	27 (0.9)	1 (0.0)	29 (0.9)	31 (1.0)
No	3,054 (98.0)	610 (19.6)	2,041 (65.5)	403 (12.9)	308 (9.9)	1,560 (50.1)	1,186 (38.1)
Delivery model^^b^^							
Natural delivery	1,549 (49.7)	369 (11.8)	1,033 (33.2)	147 (4.7)	174 (5.6)	808 (25.9)	567 (18.2)
Cesarean delivery	1,566 (50.3)	245 (7.9)	1,038 (33.3)	283 (9.1)	135 (4.3)	781 (25.1)	650 (20.9)
Gestational age (weeks)^a,b,d^	39.4 (38.8–40.1)	39.6 (39.0–40.3)	39.43 (38.9–40.1)	39.42 (38.7–40.0)	39.43 (38.7–40.0)	39.43 (38.7–40.1)	39.43 (39.0–40.3)
Birth weight(g)^a,b,c^	3357.5 (390.6)	3249.2 (348.9)	3362.3 (385.2)	3488.8 (428.0)	3203.2 (361.5)	3321.9 (371.4)	3443.1 (403.0)
Gender							
Male	1,620 (52.0)	312 (10.0)	1,082 (34.7)	226 (7.3)	159 (5.1)	836 (26.8)	625 (20.1)
Female	1,495 (48.0)	302 (9.7)	989 (31.7)	204 (6.5)	150 (4.8)	753 (24.2)	592 (19.0)
MDI scores^a,c^	107.97 (22.22)	108.41 (21.91)	108.37 (22.14)	105.42 (22.92)	109.69 (23.29)	108.43(22.25)	107.70(21.91)
PDI scores	107.83 (18.34)	107.48 (17.65)	107.92 (18.50)	107.93 (18.59)	108.72 (19.42)	107.71 (18.90)	107.77 (17.30)

BMI, prepregnancy body mass index; GWG, gestational weight gain; GDM, gestational diabetes mellitus; HDP, hypertensive disorders of pregnancy; MDI, Mental Developmental Index; PDI, Psychomotor Developmental Index.

^a^ *P* < 0.05 difference by analysis of *χ*^2^ of prepregnancy BMI category.

^b^ *P* < 0.05 difference by analysis of *χ*^2^ of gestational weight gain category.

^c^ The data were presented as mean(standard deviation).

^d^ The data were presented as median (25th–75th).

We first want to determine if maternal pre-pregnancy BMI groups and GWG groups are associated with baby infants’ MDI and PDI scores in our multivariable models. Table 2 displays the results of the association for the adjusted and unadjusted linear regression.

**Table 2:** Correlations among child neurodevelopment scores at age 2 and PBMI and GWG categories.

	Underweight (*n**** ***= 614)	Normal weight (*n**** ***= 2,071)	Overweight/Obese (*n**** ***= 430)	Inadequate (*n**** ***= 692)	Adequate (*n**** ***= 1,646)	Excessive (*n**** ***= 473)
MDI, *β* (95%CI)						
Unadjusted	0.044 (−1.957, 2.044)	0.00 (R)	−2.954 (−5.261, −0.646)	−1.744 (−4.453, 0.965)	0.00 (R)	−0.734 (−2.393, 0.926)
Adjusted^a^	0.875 (−1.261, 3.010)	0.00 (R)	−2.479 (−4.800, −0.158)	−2.120 (−4.940, 0.700)	0.00 (R)	−0.874 (−2.591, 0.844)
Adjusted^b^	0.929 (−1.201, 3.059)	0.00 (R)	−2.510 (−4.821, −0.200)	−1.998 (−4.782, 0.785)	0.00 (R)	−1.042 (2.773, 0.688)
PDI, *β* (95%CI)						
Unadjuste	−0.434 (−2.087, 1.219)	0.00 (R)	0.014 (−1.893, 1.920)	1.014 (−1.222, 3.251)	0.00 (R)	−0.142 (−1.727, 2.010)
Adjusted^a^	−0.824 (−2.592, 0.945)	0.00 (R)	0.239 (−1.683, 2.161)	1.270 (−1.065, 3.606)	0.00 (R)	0.604 (−0.818, 2.026)
Adjusted^b^	−0.893 (−2.656, 0.909)	0.00 (R)	0.381 (−1.553, 2.315)	1.425 (−0.905, 3.754)	0.00 (R)	0.589 (−0.860, 2.037)

PBMI, prepregnancy body mass index. GWG, gestational weight gain categories. CI, confidence interval; OR, odds ratio; MDI, mental development index; PDI, psychomotor development index.

^a^ Adjusted for maternal age, parity, maternal education, GWG, GDM, HDP, family yearly income and passive smoking during pregnancy.

^b^ Adjusted for maternal age, parity, maternal education, GWG, GDM, HDP, family yearly income, passive smoking during pregnancy, delivery mode, gender, gestational age and birth weight.

*P* for interaction = 0.246 for MDI; *P* for interaction = 0.869 for PDI.

The appropriate GWG or normal pre-pregnancy BMI was employed as the reference in all regression model outcomes.

The relationship among pre-pregnancy BMI classes, GWG, and newborn neurobehavioral progress is shown in Table 2. The MDI scores of newborns of overweight and obese mothers were lower than the adjusted MDI scores of infants of normal-weight moms after controlling for maternal age, parity, maternal education, GWG, GDM, HDP, family yearly income, and passive smoking while pregnant. (*β *= −2.479, 95%CI*** ***= −4.800 to −0.158). For delivery method, gender, gestational age, and birth weight, we made additional adjustments. The relationship was still significant (*β *= −2.510, 95%CI*** ***= −4.821 to −0.200). Infant MDI scores and mother GWG categories did not show any statistically meaningful correlations. Infants PDI scores did not substantially differ according to the maternal prepregnancy BMI and GWG groups.The interaction term (BMI × GWG) was also explored by introducing into a generalized linear equation.The result showed that the interaction effect was not significantly associated with MDI (*P *= 0.246) or PDI (*P *= 0.869). As a result, in none of our sensitivity studies did we take the interaction into account. Then, in each pre-pregnancy BMI category subgroup, stratification analyses and linear regressions were carried out to investigate the association between GWG and neurodevelopment. In comparison to the referred sufficient GWG women, inadequate GWG was highly related to the reduced MDI scores in mothers with normal maternal pre-pregnancy BMI (*β* = −3.952, 95%CI = −7.809 to −0.094). (Table 3). Infants of high GWG moms had reduced MDI scores (*β* = −5.173, 95%CI = −9.803 to −0.543) in the underweight pre-pregnancy BMI mothers (Table 3). The PDI scores, in contrast, were not affected by the mother’s pre-pregnancy BMI or GWG.

**Table 3:** Stratified by pre-pregnancy BMI categories, infantile neurodevelopment at 2 years of age was compared in the various GWG categories.

	MDI			PDI		
	***β***	95%CI	***P***	***β***	95%CI	***P***
Underweight						
Inadequate weight gain	−2.515	−7.751, 2.721	0.346	0.328	−3.929, 4.616	0.880
Excessive weight gain	−5.173	−9.803, −0.543	0.029	−0.825	−4.616, 2.966	0.669
Normal						
Inadequate weight gain	−3.952	−7.809, −0.094	0.045	1.284	−1.970, 4.539	0.439
Excessive weight gain	−0.215	−2.279, 1.850	0.172	0.500	−1.242, 2.242	0.574
Overweight/Obese						
Inadequate weight gain	−0.660	−10.556, 9.235	0.231	3.027	−5.147, 11.200	0.467
Excessive weight gain	−0.790	−5.559, 3.979	0.745	2.525	−1.414, 6.464	0.208

PBMI, prepregnancy body mass index. CI, confidence interval; MDI, mental development index; PDI, psychomotor development index.

Maternal age, parity, maternal education, pre-pregnancy BMI, GDM, HDP, family yearly income, passive smoking during pregnancy, delivery mode, gender, gestational age and birth weight were used as covariates.

All the results of regression models used the normal pre-pregnancy BMI or adequate GWG as a reference.

Table 4 provides an overview of the sensitivity analysis findings. The relevance and course of the results is changed when an alternative GWG and BMI standard is used. There were no associations between maternal GWG categories and infant MDI or PDI scores.

**Table 4:** Compares infant mental neurodevelopment at age 2 in accordance with the various IOM GWG categories according to wHO pre-pregnancy BMI classifications.

	MDI			PDI		
	***β***	95%CI	***P***	***β***	95%CI	***P***
Underweight						
Inadequate weight gain	−1.737	−6.779, 3.306	0.499	0.811	−3.309, 4.930	0.699
Excessive weight gain	−3.631	−7.503, 0.240	0.066	−1.680	−4.843, 1.483	0.297
Normal						
Inadequate weight gain	−1.093	−3.809, 1.622	0.430	1.819	0.466, 4.103	0.119
Excessive weight gain	0.695	−1.353, 2.743	0.506	1.023	−0.700, 2.746	0.244
Overweight/Obese						
Inadequate weight gain	−0.169	−10.260, 9.922	0.974	2.152	−6.180, 10.484	0.611
Excessive weight gain	−0.724	−6.50, 5.052	0.805	2.249	−2.521, 7.018	0.354

PBMI, prepregnancy body mass index. GWG, gestational weight gain categories. CI, confidence interval; MDI, mental development index; PDI, psychomotor development index.

Maternal age, parity, maternal education, pre-pregnancy BMI, GDM, HDP, family yearly income, passive smoking during pregnancy, delivery mode, gender, gestational age and birth weight were used as covariates.

All the results of regression models used the normal pre-pregnancy BMI or adequate GWG as a reference.

## Discussion

A substantial correlation among maternal BMI, GWG, and neurobehavioral developmental variability at 2 years of age was found in this nationally representative sample of infants under 2 years of age. Infants of moms with an abnormal GWG or an overweight/obese prenatal BMI showed substantial negative connections with their cognitive development but did not affect their psychomotor development. More specifically, inadequate GWG was linked to poorer MDI scores for moms who were of normal weight before conception. Babies born to underweught mothers with high GWG levels exhibited more delayed cognitive development. The mean MDI score of babies born to obese or overweight moms was a little lower than that of babies born to mothers who were of a healthy weight. The study’s findings concur with our theory. The standardised prevalence of obesity in China was 8.1% in 2018, more than twice as high as in 2004 (30), as a result of the country’s current obesity pandemic. It is necessary to emphasise the significance of maternal pre-pregnancy BMI and GWG data for newborn neurobehavioral development.

There has been some recent research on the association between mother pre-pregnancy BMI and/or GWG and infants’ neuro development. Similar to our findings, a study in Shanghai, China, infants aged 1 year revealed that mothers who were overweight/obese before becoming pregnant had infants that were lacking in neurobehavioral development, including linguistic capacity and social conduct. Women with high GWG, however, only exhibited a decline in social behavior (31). Although we were unable to look at connections between the various cognitive domains in this study. Insufficient maternal GWG may harm an infant’s neurodevelopment, according to a large survey conducted in Japan in 2021 (32). GWG below recommended levels was linked to a significantly higher risk of developmental delay for the domains of communication (odds ratio [OR] 1.21, 95% confidence interval [CI] 1.09–1.34), gross motor (OR 1.14, 95%CI, 1.05–1.24), fine motor (OR 1.13, 95%CI, 1.04–1.24), problem-solving (OR 1.09, 95%CI, 1.01–1.18), and personal-social (OR 1.15, 95%CI, 1.08–1.25) (32). Contrary to these research, a correlation between maternal obesity or overweight and a child’s cognitive development was not found in two cohorts of European infants at around 3 years of age (32, 33). That study had a drawback, though, in that the evaluation of newborns’ cognitive ability was dependent on subjective maternal reports as opposed to impartial tests. The following contradictory results could be explained by many variables. First off, the linear regression models used in the majority of these studies can hide the true nonlinear relationship between prenatal body mass and a child’s intellectual abilities. Second, some investigations may not have used the best metrics of maternal prepregnancy BMI or child neurodevelopment (34, 35). Small sample sizes may also reduce the statistical power of certain studies (36).

The relationship between a mother’s pre-pregnancy BMI and her child’s intellectual growth has been noticed, and numerous mechanisms may be involved. Inadequate prenatal micronutrient status (37–40), high-stress levels (41–43), and an inflammatory intrauterine environment (44–46) are a few potential biological causes. Particularly, obesity-related intrauterine inflammation may have a direct impact on the cognitive feature of offspring by harming the developing foetal brain (45, 46) or by continuing to increase the permeability of the foetal blood-brain barrier (47), making the developing foetus more susceptible to other intrauterine toxic agents. Why insufficient GWG might lead to neurodevelopmental problems is unknown. Malnutrition may limit embryonic brain growth, which is one explanation. Since specific nutrients are needed at delicate or crucial stages of foetal development, the quality of the mother’s food is crucial (48). Folic acid is known to be essential for neural tube development (49). Since myelination, the formation of the frontal cortex, and the basal ganglia all depend on iron, iron insufficiency is the most typical nutritional shortfall throughout pregnancy (50). Sussman et al. hypothesised that early exposure to a carbohydrate-restricted diet, like the currently well-liked ketogenic diet programmes, affected offspring neuroanatomy, such as brain structure and volumetric change (51), as well as behavioural alterations, such as decreased susceptibility to anxiety and depression and increased hyperactivity in adult mouse offspring (51). Contrarily, excessive prenatal weight gain may worsen the already high levels of inflammation linked to obesity (52, 53), impeding development (50–54). For underweight women of childbearing age, it would seem crucial to follow the best diet and weight-gain advice.

The development of an infant’s motor skills at age 2 was not shown to be correlated with the maternal pre-pregnancy BMI or GWG categories. Young newborns’ motor development is relatively resistant to mild influences, and the ages at which healthy infants complete developmental goals in this area vary widely. As a result, utilising broad-based tests, it may be challenging to identify population-level assessments of dedicated motor development (55). Long-term motor results should therefore be further studied in the future.

The study, one of the largest probable birth cohort studies in the middle of China, was meticulously carried out with a high rate of long-term follow-up to determine the effect of maternal exposures on child health. In this study, professional psychologists assessed the neurodevelopment of more than 3,000 infants at the age of two using highly standardised methods, a detailed protocol, and rigorous quality control measures. These advantages made the research especially ideal for researching the relationship between the BMI and GWG of the mother before conception and the neurodevelopment of the foetus. The high sample size gave us enough statistical power to investigate the effects of possible confounders including hypertensive diseases and gestational diabetes. In contrast to earlier findings, our study embraced the Life Cycle Project-Maternal Obesity and Childhood Outcomes Study Group’s optimal GWG ranges guideline in 2019 but not IOM. And a significant association between abnormal GWG and infants’s mental development was successfully found compared with the IOM GWG guidelines. According to this study, not only in China but also in other developed nations, it is necessary to reevaluate the ideal pre-pregnancy BMI and GWG for women who want to become pregnant. To support this assertion, more research with a larger sample size is required.

There are several restrictions to this study. First, because our sample size was so small, we were unable to conduct additional BMI subgroup analyses, such as those for overweight and obese people. Second, self-reported data on mother weight during pregnancy, as in the majority of studies in this area, may have skewed results. The information for this investigation was then obtained from a single clinic. Further research is necessary to determine how the reported findings affect external validity, even if they may reflect the features of the mother-infant group in this region. We lacked information on postnatal environmental factors, which are also crucial for infants’ neurobehavioral development (56, 57). Studies examining correlations of prenatal exposure levels with offspring healthcare outcomes are susceptible to residual confounding by genetic and environmental factors that are shared within families. Future observational studies examining the causative role of maternal prepregnancy BMI and GWG in offspring’s neurodevelopmental outcomes must take into account any potential residual contamination by this shared familial variables (58).

We discovered that abnormal GWG and high pre-pregnancy BMI were both linked to impaired cognitive development in 2-year-old infants. Our findings imply that babies born to inadequate or excessive GWG moms have lower MDI scores and may consequently need greater monitoring for developmental impairments, even if the exact mechanism is still unknown. A healthy weight and lifestyle before getting pregnant should be promoted to all women of reproductive age, according to this study. Although there are obstacles to such preconception weight loss, including a lack of knowledge and understanding, time restraints, and affordability (59), the current public health objective in China is to raise the percentage of women who are in a healthy weight range when they conceive. Future studies should look at how pregnant weight increase patterns and higher pre-pregnancy BMI values affect the cognitive development of children.

## Conclusion/future directions

For Chinese babies aged 2 in this nationally representative sample, aberrant pre-pregnancy BMI and GWG can impair infants’ mental development, but not psychomotor development. Such results are significant given the incidence of overweight and obesity as well as the long-term effects of early brain development. Additionally, women should be given general advice on how to achieve their ideal pre-pregnancy BMI and GWG. Optimal GWG recommendations proposed by 2019 Life Cycle Project-Maternal Obesity and Childhood Outcomes Study Group were more suitable for Chinese women than 2009 Institute of Medicine(IOM) guidelines. Besides, stratifying the further study by sex (male vs. female) would also strengthen to provide highly relevant and meaningful clinical information on infants’ predisposition to MDI or PDI outcomes.

## Data Availability

The original contributions presented in the study are included in the article/Supplementary Materials, further inquiries can be directed to the corresponding author.
